# Effects of Irritability of the Youth on Subjective Well-Being: Mediating Effect of Coping Styles

**DOI:** 10.18502/ijph.v49i10.4685

**Published:** 2020-10

**Authors:** Ling ZHANG, Benxian YAO, Xiaodan ZHANG, Hao XU

**Affiliations:** 1. Center for Mental Health, Shaoxing University, Shaoxing, China; 2. College of Educational Science, Anhui Normal University, Wuhu, China; 3. College of Teacher Education, Hefei Normal University, Hefei, China

**Keywords:** Youth, Irritability, Coping style, Subjective well-being

## Abstract

**Background:**

The global COVID-19 pandemic caused great impacts and influences to human psychology. As a result, youths who are kept at home for a long time easily develop irritability and problematic behaviors. However, relatively little attention has been paid to the relations among irritability, coping style, and subjective well-being of the youth.

**Methods:**

Overall, 1,033 youth respondents (aged 18–30 yr) from seven provinces in China were investigated in 2020 using the irritability, depression, and anxiety scale, coping style scale, and well-being index scale.

**Results:**

Among the dimensions of irritability of the youth, anxiety received the highest score, followed by introversion irritability, extroversion irritability, and depression. Irritability had significant regional differences. The total score of irritability among rural youth was significantly higher than that of urban youth (*P<*0.05). The irritability level of youths with parents’ emotional status was harmonious and good relations with family members and peers was far lower than those of youths who have poor relations between parents, family members, and peers (*P<*0.05). The irritability level of youths with a lower monthly household income was higher (*P<*0.05). Irritability of the youth had significantly negative correlations with positive response and SWB, and it had a significantly positive correlative with negative response. Coping style can mediate the relationship between irritability and SWB of the youth to some extent.

**Conclusion:**

Significant correlations exist among irritability, coping style, and SWB of the youth. Irritability can be used to predict SWB indirectly through positive response.

## Introduction

Irritability is the expression of anger of individuals upon setbacks. It is characterized by continuous anger, grouchiness, depression, or raging outbursts in a short period. Overreaction to stimuli is the major characteristic of irritability (1–2). Given that mood disorder is the main clinical symptom of irritability, the World Health Organization has added irritability to the Diagnostic and Statistical Manual of Mental Disorders (5th ed) to provide better understanding of the public health needs brought about by the disorder (3). In fact, irritability is a trait that manifests throughout character development of the youth (4). A survey on approximately 200,000 youth respondents from different countries reported that 10.7% of youth had irritability behaviors (5). Irritability has become an important factor that influences the psychological health of the youth, and due to its frequent occurrence and serious consequences on psychological health of the youth, it has attracted extensive attentions from scholars (6).

The global COVID-19 pandemic, which began in January 2020, has caused high levels of panic, anxiety, worry, and depression. To prevent the spread of the COVID-19 pandemic, the Ministry of Education of the People’s Republic of China postponed the spring semester of 2020. As a result, youths who are kept at home for a long time easily develop irritability and problematic behaviors. Therefore, discussing the consequences of irritability of the youth is vital in the prevention of and intervention to psychological health issues of the youth.

Irritability is closely related with subjective well-being (SWB). Individuals with an irritability tendency mainly show social adaptation disorder and mood disorder (7–8). Therefore, the SWB of the youth can be predicted to some extent from irritability (9). According to a survey, 75% of 130 juvenile delinquents (aged 13.8–19.5) in custody have irritability and low SWB (10). According to the Centers for Disease Control and Prevention (USA) in 2017, 23.6% of 9th to 12th Grade students had been reported at least one irritability behavior of physical aggression in the past years, and these respondents showed poor interpersonal relations (11). Irritability not only influences living quality and well-being experience of the youth, but it is also related with depression, anxiety, and suicide in adulthood (12). Irritability is clearly a critical state. Despite the absence of evident external characteristics, individuals’ control over temper is weakened, resulting in an outburst of bad behaviors, such as grumpiness and vituperation. These behaviors may be either temporary or long-term, and they are unpleasant experiences of individuals. They influence the SWB level of individuals. In the present study, irritability is used as an observation variable of the SWB of the youth.

Irritable youths easily generate avoidance behavior and aggressive behavior, because they are dissatisfied with practical life (13). According to a tracking survey, irritability easily causes antagonism disorder of the youth (14), and youths with irritability tendency exhibit disregard for social order, negative response strategies, and frequent destructive behaviors (15). These behaviors can influence their SWB experiences. Thus, irritability not only influences SWB of individuals directly, but it can also act indirectly through other factors. Coping style refers to conscious, purposeful, and flexible adjustment behaviors that individuals make to adapt to practical environmental changes. Coping style of individuals can influence SWB experiences. Therefore, a positive coping style is conducive to improving SWB experiences, whereas a negative coping style will intensify setback experiences and thereby lower SWB (16). Moreover, coping style can relieve the relationship between perfectionism and risk of suicide among the youth (17). Youths who hardly use coping strategies have relatively low SWB level (18). Therefore, irritability is a common psychological problem of the youth, and it influences their psychological health and SWB index. Furthermore, influences of irritability on SWB of the youth may not be direct, but may be indirect through coping styles.

In the present study, irritability issues of the youth and relevant differences were investigated. Internal relations among irritability, coping style, and SWB of the youth were analyzed. Research conclusions provide schools, communities, and society with references to prevent and intervene in irritability issues of the youth, thus improving the level of SWB.

## Methods

### Research tools

Irritability depression and anxiety scale was compiled by Snaith (19). It involves 18 items, and each item was scored from level 0 to level 3. Four dimensions were assessed, including depression, anxiety, introversion irritability, and extroversion irritability. A higher score reflects more serious emotional problems. Each dimension was divided into three degrees, namely, normal state, critical state, and abnormal state. The coefficient of internal consistency (Cronbach’s α) of four dimensions in the IDA scale (Chinese version) ranged between 0.419–0.769. The Cronbach’s α of this questionnaire was 0.845.

Simplified ways of coping questionnaire: the simplified questionnaire of coping style was compiled by Xie (20). It contains 20 questions arranged in 4 levels from “no use” to “frequent use.” The first 12 questions were used to measure positive response level of respondents and the remaining 8 questions were used to measure negative response level of respondents. The Cronbach’s α of this questionnaire was 0.769.

SWB scale: the SWB scale compiled by Campbell, which was translated into Chinese (21). This scale covers an overall emotional index scale composed of 8 items (each item was scored in 7 levels and described emotional connotations from different perspectives) and a questionnaire on life satisfaction composed of only one question. The Cronbach’s α of this questionnaire was 0.955.

### Data Collection

Youth respondents were selected by stratified cluster random sampling according to economic status in different regions of China. Respondents were from Zhejiang, Fujian, and Jiangsu in Eastern China; Anhui and Henan in Central China; and Shanxi and Guangxi in Western China. Data collection was conducted in 2020. In light of the COVID-19 pandemic, data were collected through an online questionnaire survey. A total of 1,100 questionnaires were sent, and 1,033 questionnaires were collected (aged 18–30), showing an effective collection rate of 93.9%. Specifically, 412 respondents were from Eastern China (39.9%), 385 respondents were from Central China (37.3%), and 236 respondents were from Western China (22.8%). A total of 450 respondents were male (43.5%) and 583 were female (56.5%). Among them, 585 were university students (56.6%) and 448 were workers (43.4%). The sample was composed of 625 urban youth (60.5%) and 408 rural youth (39.5%).

Data processing and analysis were conducted using SPSS 21.0. Irritability, coping style, and assessment results of the SWB scale of respondents were analyzed. Irritability status and difference, as well as relations among irritability, coping style, and SWB of the youth were investigated through *t*-test, one-way analysis of variance, correlation analysis, and multivariate regression analysis. Informed consent was taken from the participants before the study and the study was approved by local Ethics Committee.

## Results

### General irritability state and difference of the youth

General status and difference of respondents were calculated according to scores of the irritability scale. Tables 1 to 6 show the results. The *t*-test based on independent samples was applied in the case of two variables, and the variance analysis was adopted for more than two variables.

**Table 1:** Descriptive statistics of irritability on all respondents and according to gender

***Factors***	***Scores***	***Male (n=450)***	***Female (n=583)***	***t-value***
Irritability	46.36±7.09	46.07±7.18	46.50±7.05	−0.904
Depression	10.28±1.98	10.42±2.12	10.22±1.91	1.516^*^
Anxiety	13.53±2.41	13.27±2.47	13.65±2.38	−2.314
Introversion irritability	11.83±2.39	11.71±2.33	11.88±2.42	−1.107
Extroversion irritability	10.71±2.02	10.66±1.96	10.73±2.05	−0.585

* *P*<0.05,

** *P*<0.01,

*** *P*<0.001

**Table 2:** Influences of family location on irritability of the youth

***Variable***	***Urban area***	***Rural area***	***t-value***
Irritability	46.15±7.48	46.53±6.78	−0.823^*^
Depression	10.18±2.07	10.36±1.91	−1.472
Anxiety	13.35±2.48	13.66±2.35	−2.041
Introversion irritability	11.86±2.53	11.80±2.28	0.420^**^
Extroversion irritability	10.75±2.14	10.68±1.92	0.494^*^

* *P*<0.05,

** *P*<0.01,

*** *P*<0.001.

**Table 3:** Effects of parents’ emotional status on irritability of the youth

***Variable***	***Good***	***Moderate***	***Poor***	***F***
Irritability	44.54±6.85	48.55±6.49	49.71±7.46	−50.354^***^
Depression	9.70±1.80	11.00±1.90	11.24±2.14	−66.289^***^
Anxiety	13.00±2.39	14.03±2.30	14.32±2.45	−22.292^***^
introversion irritability	11.32±2.31	12.39±2.26	12.98±2.57	−34.857^***^
extroversion irritability	10.40±1.92	11.11±2.03	11.15±12.29	−16.168^***^

* *P*<0.05,

** *P*<0.01,

*** *P*<0.001.

**Table 4:** Influences of relationship with family members on irritability of the youth

***Variable***	***Good***	***Moderate***	***Poor***	***F***
Irritability	44.97±6.65	50.02±6.58	58.63±6.56	−74.420^***^
Depression	9.86±1.81	11.42±1.95	12.90±1.75	−79.298^***^
Anxiety	13.20±2.34	14.37±2.31	16.90±2.62	−35.566^***^
Introversion irritability	11.47±2.30	12.75±2.33	15.27±2.14	−41.646^***^
Extroversion irritability	10.42±1.88	11.46±2.13	13.54±2.69	−38.862^***^

* *P*<0.05,

** *P*<0.01,

*** *P*<0.001.

**Table 5:** Influences of relationship with peers on irritability of the youth

***Variable***	***Good***	***Moderate***	***Poor***	***F***
Irritability	44.28±6.58	49.52±6.54	61.00±4.54	−87.415^***^
Depression	9.799±1.87	11.02±1.89	13.75±1.50	−58.267^***^
Anxiety	12.97±2.30	14.37±2.30	18.00±2.44	−52.190^***^
Introversion irritability	11.27±2.26	12.68±2.31	15.25±2.87	−51.079^***^
Extroversion irritability	10.23±1.83	11.43±2.07	14.00±2.30	−52.817^***^

* *P*<0.05,

** *P*<0.01,

*** *P*<0.001.

**Table 6:** Influences of monthly household income on irritability of the youth

***Variable***	***1,500–3,000***	***3,100–4500***	***4,600–6,000***	***>6,000***	***F***
Irritability	47.81±6.77	47.12±6.54	46.91±7.83	45.06±6.87	8.396^***^
Depression	10.72±1.98	10.49±1.94	10.58±2.00	9.82±1.90	13.102^***^
Anxiety	14.09±2.26	13.87±2.32	13.60±2.56	13.07±2.37	9.608^***^
Introversion irritability	12.18±2.49	11.89±2.21	11.97±2.53	11.57±2.34	3.058^*^
extroversion irritability	10.80±1.96	10.86±2.05	10.74±2.15	10.57±1.95	1.164

* *P*<0.05,

** *P*<0.01,

*** *P*<0.001.

Table 1 shows that the mean score of irritability of the youth was (46.36±7.09). The mean of each question is 2.57, which is higher than the theoretical median (2.55). Hence, the general irritability of respondents is at a critical state. Specifically, anxiety has the highest score (13.53±2.41), followed by introversion irritability (11.83±2.89), extroversion irritability (10.71±2.02), and depression (10.28±1.98), successively. The general irritability of female respondents is higher than that of male respondents, but the difference is not significant. Male respondents showed a significantly higher score in depression than female respondents (*P<*0.05). Female respondents showed higher total scores in irritability, anxiety, introversion irritability, and extroversion irritability than male respondents, but the differences are not significant.

Table 2 shows a significant regional difference in irritability of the youth. Rural youths have a significantly higher total score of irritability than urban youths (*P<*0.05). Furthermore, regional differences on the four dimensions of irritability were tested. Urban youths show significantly higher scores in introversion irritability and extroversion irritability than rural youths (*P<*0.05), but rural youths show slightly higher scores in depression and anxiety (Table 2).

Table 3 shows that parents’ emotional status has extremely significant influences on irritability of the youth (*P<*0.001). Youths show a lower irritability level when the parents’ emotional status is good. Specifically, respondents with a better relationship between parents show a significantly lower irritability level than those with a poor relationship between parents. The relationship between parents can cause extremely significant influences on depression, anxiety, introversion irritability, and extroversion irritability of the youth. Youths from families with good relationship between parents show significantly lower scores in depression, anxiety, introversion irritability, and extroversion irritability than those from families with poor relationship between parents (*P<*0.001).

Table 4 shows that relationship with family members can cause extremely significant influences on irritability of the youth. Youths who have good relationship with family members have extremely significantly lower irritability level than youths who have poor relationship with family members (*P<*0.001). Relation with family members has extremely significant influences on depression, anxiety, introversion irritability, and extroversion irritability. Youths who have good relationship with family members show significantly lower scores in depression, anxiety, introversion irritability, and extroversion irritability than youths who have poor relationship with family members (*P<*0.001).

Table 5 shows significant influences of relationship with peers on irritability of the youth. Youths who have good relations with peers show extremely significantly lower irritability level than youths who have poor relations with peers (*P<*0.001). Extremely significant differences were found in depression, anxiety, introversion irritability, and extroversion irritability of youths who have poor relations with peers. Specifically, youths who have good relationship with peers show significantly lower scores in depression, anxiety, introversion irritability, and extroversion irritability than youths who have poor relationship with peers (*P<*0.001).

Table 6 shows that monthly household income has extremely significant influences on irritability of the youth (*P<*0.001). The higher the monthly household income is, the lower irritability of the youth will be. Moreover, depression, anxiety, and introversion irritability of the youth vary significantly with monthly household income. Youths with higher monthly household income have lower scores in depression, anxiety, and introversion irritability (*P<*0.05). However, monthly household income cannot influence extroversion irritability significantly.

### Relations among irritability, coping style, and SWB of the youth

Table 7 shows the results and calculations of the general conditions of respondents according to the scores in the irritability scale, coping style scale, and SWB scale.

**Table 7:** Relations among irritability, coping style, and SWB of the youth

***Variable***	***Irritability***	***Positive coping***	***Negative coping***	***SWB***
Irritability	1			
Positive coping	−0.48^**^	1		
Negative coping	0.26^**^	−0.10^**^	1	
SWB	−0.64^**^	0.45^**^	−0.17^**^	1

* *P*<0.05,

** *P*<0.01,

*** *P*<0.001.

Table 7 shows that the total score of irritability of the youth has significantly negative relations with positive coping and SWB (*P<*0.01), with correlation coefficients of −0.48 and −0.64, respectively. Irritability of the youth has a significantly positive correlation with negative coping (*P<*0.01), and the correlation coefficient is 0.26. A significantly negative correlation exists between SWB and negative coping of the youth (*P<*0.01), and the correlation coefficient is −0.17. SWB and positive coping of the youth have a significantly positive correlation (*P<*0.01), and the correlation coefficient is 0.45. Thus, SWB level is negatively related with irritability level, and different coping styles can influence the SWB experience of the youth.

### Mediating effect test of coping style in the relationship between irritability and SWB of the youth

The analysis on relations among irritability, positive coping, negative coping, and SWB of the youth reveals significantly correlations among them (*P<*0.01). This result makes the testing of the mediating effect of coping style possible. Hence, this study analyzed the mediating effect of positive coping and negative coping on the relationship between irritability and SWB. The analysis was done through regression analysis to disclose relations among different variables. Moreover, an OLS multivariate regression test was carried out by using irritability as an independent variable, positive coping and negative coping as mediating variables, and SWB as a dependent variable.

Multilayer regression analysis method was applied, and negative coping did not involve into the regression equation. Therefore, this study only analyzed the mediating effect of positive coping on the relationship between irritability and SWB. Table 8 shows the results. Irritability of the youth has a significantly negative prediction effect to SWB (*β*=−0.63 and *P<*0.001). In addition, irritability of the youth has a significantly negative prediction effect on positive coping (*β*=−0.47, *P<*0.001). After positive coping is added, irritability (*β*=−0.54, *P<*0.001) and positive coping (*β*=0.18, *P<*0.001) have a significant prediction effect on SWB. Positive coping is a mediating variable between irritability and SWB of the youth. Thus, irritability can influence SWB through the mediating variable of positive coping. The regression coefficient of irritability (*β*=−0.54, *P<*0.001) reflects that positive coping can mediate the relationship between irritability and SWB of the youth to some extent. The ratio between the mediating effect and the overall effect is 13.42%. Therefore, the mediating effect of positive coping is 0.13.

**Table 8:** Mediating effect of coping style in the relationship between irritability and SWB

***Steps***	***Dependent variables***	***Independent variable***	***R^2^***	***Adjusted R^2^***	***F-value***	***β***	***t***
1	SWB	Irritability	0.40	0.40	704.93^***^	−0.63	−26.55^***^
2	Positive coping	Irritability	−0.41	0.22	307.08^***^	−0.47	−17.52^***^
3	SWB	Irritability				−0.54	−20.48^***^
		Positive coping	0.43	0.43	393.71^***^	0.18	7.02^***^

* *P*<0.05,

** *P*<0.01,

*** *P*<0.001.

## Discussions

According to the analysis on overall irritability level of the youth in Table 1 and Table 2, the overall irritability of the youth is at a critical level. The relatively high irritability level of the youth is related with not only their psychological qualities, but also with the social environment. Female youth generally show higher irritability level than male youth, but the difference is not significant. In contrast, male respondents have higher scores in depression. This result reflects gender differences in irritability (22). Influences of genetic factors on irritability of male youth increase slightly over time, whereas influences of genetic factors on irritability of female youth decrease to some extent (23). The youth have weaker psychological capacity than other age groups, and they are highly sensitive to emotional damages. The youth bear many pressures from academic study, employment, postgraduate entrance exams, and daily life. As these concerns accumulate, they need to be vented, which is mainly manifested by instable emotions and high sensitivity to stimuli threshold. Therefore, the youth easily develop irritability. Significant regional differences exist in overall irritability of the youth. Specifically, the total score of irritability of rural youth is significantly higher than that of urban youth. Furthermore, regional differences on the four dimensions of irritability were tested, revealing that urban youth showed significantly higher scores of introversion irritability and extroversion irritability than rural youth. This finding may be because most urban youths are only child, and they receive more affection from their parents. As a result, they have poor resistance to setbacks and lack the ability to live independently.

According to the results in Tables 3 to 6, parents’ emotional status, relationship with family members, and relationship with peers have significant impacts on irritability level of the youth. Youths who have good relationship between parents, good relationships with family members, and good relationships with peers show the lowest irritability level. These findings prove that relationship between parents, relationship with family members, and relationship with peers are key influencing factors of irritability of the youth. Relationship between parents can influence continuous development of irritability of the youth (24). Rage, depression, and education behaviors of parents are related with rage, aggressive behaviors, and extroversion problems of children (25). In adolescence, relationship with peers becomes increasingly important. For example, experiences in social exclusion and risk factors and emotions (e.g., social damages and irritability) will often cause psychological anxiety (26). Monthly household income has extremely significant impacts on irritability level of the youth. The irritability level of the youth is negatively correlated with monthly household income. SWB increases with the improvement of material standard of living. In particular, monthly household income or changes in family structure can influence the relationship between parents and children when the per capita income is at a relatively low level (27).

The results in Table 7 and 8 indicate significant correlations among irritability, coping style, and SWB of the youth. Individuals with higher irritability level have negative cognition and strong aggression. They often make physical and verbal attacks upon stimuli. However, positive response strategy can relieve irritability of the youth (28).

SWB can be predicted from irritability level and positive coping. According to the changes in regression coefficient of irritability, positive coping can mediate the relationship between irritability and SWB.

On the basis of these results, insights on intervention efforts could be gained: first, Irritability emotions and behaviors of the youth can be improved, and cognitive assessment can be adjusted. In other words, cognitive assessment of anxiety on damages to psychological health is decreased. Second, training cognitive reassessment refers to providing cognitive assessment on proper benefits of anxiety to psychological health. For example, fatigue and irritability can be relieved by prolonging sleep duration (29). Moreover, the cognition mode of the youth is improved, and positive coping is adopted to improve SWB of the youth. During the pandemic, youths who are kept at home while studying and working are suggested to communicate and interact with family members, friends, and neighbors. This suggestion can be a feasible public health strategy, and it can also encourage the youth to seek professional psychological or metal help.

## Conclusion

The irritability of the youth is at a critical level during a pandemic. Such irritability is manifested by serious anxiety. Moreover, irritability of the youth is significantly correlated with family location, parents’ emotional status, relationship with family members, relationship with peers, and monthly household income. Irritability of the youth not only can influence SWB directly, but it can also influence SWB indirectly through positive coping.

## Ethical considerations

Ethical issues (Including plagiarism, Informed Consent, misconduct, data fabrication and/or falsification, double publication and/or submission, redundancy, etc.) have been completely observed by the authors.
